# Carving the Future: Career Aspirations of Senior Dental Students in Saudi Arabia

**DOI:** 10.1055/s-0045-1812111

**Published:** 2025-10-28

**Authors:** Mohammad Z. Nassani, Anas B. Alsalhani, Alwaleed A. Alasmari, Waleed S. Alqahtani, Samer Rastam, Enass Shamsy, Mohammed Noushad, Salah Sakka, Faisal M. Alali, Bassel Tarakji, Kamran Ali, Sadeq A. Al-Maweri

**Affiliations:** 1 Department of Restorative and Prosthetic Dental Sciences, College of Dentistry, Dar Al Uloom University, Riyadh, Saudi Arabia; 2 Department of Dentistry, Vision Colleges, Riyadh, Saudi Arabia; 3 Medical Services of Royal Saudi Land Forces, Ministry of Defense, Riyadh, Saudi Arabia; 4 Department of Dental, Medical Services - Ministry of Interior, Riyadh, Saudi Arabia; 5 Department of Medicine, Vision Colleges, Riyadh, Saudi Arabia.; 6 Department of Dental Health Care, Inaya Medical College, Riyadh, Saudi Arabia; 7 Department of Oral and Maxillofacial Surgery and Diagnostic Sciences, College of Dentistry, Prince Sattam Bin Abdulaziz University, Al Kharj, Saudi Arabia; 8 College of Dental Medicine, QU Health, Qatar University, Doha, Qatar

**Keywords:** career choice, career guidance, dental students, dentistry, Saudi Arabia

## Abstract

**Objectives:**

This study investigates the future career plans of senior dental students in Saudi Arabia and the factors influencing these plans.

**Materials and Methods:**

An online questionnaire-based survey, designed following STROBE guidelines, was conducted. Participants included final-year dental students and interns from dental institutions across Saudi Arabia. The survey collected demographic data, career preferences, and factors potentially impacting career choices.

**Results:**

A total of 584 students from 12 dental schools participated. Most respondents (63.5%) reported receiving career guidance, with 87.5% expressing interest in postgraduate studies and 11.3% preferring to work as general dental practitioners. A significant proportion (63.2%) favored employment in the government sector, while 12.8% preferred the private sector. Endodontics was the most preferred specialty (15.4%), followed by orthodontics (13.2%), periodontics (12.5%), and prosthodontics (12%). Additionally, 28.4% planned to retire before the age of 50 years. Career plans were significantly associated with gender, grade point average (GPA), and receipt of career guidance ( *p* < 0.05). Personal aspirations were the most influential factor in career decision-making, followed by the reputation of a postgraduate program and the demands of the national job market. Predictors of interest in postgraduate studies included a high GPA, graduation from a public university, and receiving career guidance from dental schools.

**Conclusion:**

Saudi dental students demonstrated diverse career aspirations, with a strong inclination toward postgraduate education and government sector employment. Given the importance of career guidance as a predictor of postgraduate interest, Saudi educational, health, and labor authorities should consider aligning students' career goals with the evolving demands of the national job market.

## Introduction

Saudi Arabia is an Arab kingdom located in the Middle East, west of Asia, occupying more than 2 million square kilometers and having a population of over 32 million. Over the last two decades, there has been a remarkable evolution and expansion of dental education in Saudi Arabia. Since 2000, the number of dental programs has reached 26 in both governmental and private universities, and this trend has been associated with increasing motivation and willingness among the Saudi student population to choose dentistry as a career. 1 2

Regardless of curriculum content, the structure of dental programs in Saudi Arabia includes a preparatory first year followed by 5 years of dental courses covering preclinical and clinical subjects, concluding with a 1-year dental internship. 3 While the Saudi Ministry of Education is responsible for overseeing educational programs in Saudi universities, the regulatory authority for managing and controlling health professions, including dentistry, is the Saudi Commission for Health Specialties (SCFHS). The responsibilities of SCFHS include conducting professional exams; licensing health professionals, such as general and specialist dentists; and registering, licensing, and recognizing residency programs in the medical, dental, and healthcare fields. 4

Observation of dental practice in Saudi Arabia reveals several trends. First, there has been a significant increase in the number of practicing dentists in the kingdom: from 786 in 1987 to 12,785 in 2014, 16,887 in 2016, and 27,181 in 2020. 4 5 6 Similarly, the dentist-to-population ratio improved from 1:8,906 in 1987, 5 to 1:2,666 in 2014, 7 1:1,880 in 2016, 6 and 1:1,288 in 2020. 4 This growth trend in the number of practicing dentists in Saudi Arabia might be attributed to the increasing number of dental graduates and the expansion of oral health services in the Saudi oral health sector. 6

Another notable trend is the growing number of Saudi dentists entering the job market and the gradual replacement of foreign dentists by Saudis across various professional ranks. 4 In previous decades, foreign dentists dominated in Saudi dental practice. Until 2017, the proportion of Saudi dentists was 22.08%. 6 However, this figure rose to 54.80% in 2020. 4

Regarding the proportion of general dental practitioners in the Saudi dental practice, it was reported to be 74.48% of the total dental workforce. 4

At the practice sector level, early statistics showed that most dental services were provided by non-Saudi dentists in both the public and private health sectors. Regarding Saudi dentists, the figures indicated that 66.35% were practicing in the public health sector. 6 Recent statistics reveal that most licensed dentists work in the private healthcare sector (66.15%) and that the private sector accommodates the majority of general dental practitioners (67.89%). In contrast, most specialist dentists in the kingdom are employed in the public health sector. 4 The capital city, Riyadh, stands out as the most popular place of work for most practicing dentists in Saudi Arabia. 4 Additionally, most Saudi dentists work in urban areas. 6 There is a notable deficiency of specialist dentists in rural areas of Saudi Arabia. 4 As regards gender distribution in Saudi dental practice, the proportion of practicing male dentists is slightly higher than that of female dentists (54.86 vs. 45.14%). 4

With the growing number of local dental graduates in Saudi Arabia and increasing competition in the job market, it is contemplated that the demand for postgraduate studies and residency programs among the new Saudi graduates is likely to increase significantly in the coming years, 1 8 which require immediate plans and actions by oral/dental health planners such as the SCFHS, Saudi dental schools, Ministry of Education, Ministry of Health, and Ministry of Labor.

The aforementioned trends highlight the current state of dental workforce employment in Saudi Arabia while also identifying several challenges. These include shortages of dental professionals, financial constraints, and inequalities in access to dental care, especially in rural areas and among persons with disabilities. 9 Additional challenges include the escalating number of dental graduates, the disproportionate distribution of the dental workforce in urban areas, and the high rate of unemployment among recent Saudi dental graduates. 4 6 These challenges call for the effective enhancement of oral health service delivery by adopting strategic planning, reforming oral health policies, and directing efforts to provide equal access to oral health services for all stakeholders in the kingdom. 9

In this context, understanding the career plans of recent dental graduates and the related factors for pursuing postgraduate education is pivotal for planning Saudi dental education, dental workforce development, and improving oral health services in the kingdom. Several studies have explored the factors that shape dental students' career decisions and postgraduate choices. For example, Halawany et al 7 found that family influence, private practice opportunities, and specific clinical interests were the main determinants of specialty choice, whereas the intellectual content of the specialty was ranked the least important. Similarly, Alrashdan et al 10 reported that socioeconomic background and social changes strongly shaped students' postgraduate aspirations, with affordability of specialty programs, reputation of the specialty, and personal desire emerging as significant determinants. In another context, Xu et al 11 found that nearly 90% of Chinese final-year dental students intended to pursue a graduate degree, with better job eligibility, higher income, and opportunities for further study being the key drivers. Moreover, passion for the subject and patient-related interests were the most influential factors guiding their specialty choice. These findings collectively underscore that postgraduate career intentions are influenced by a combination of personal interest, family and social background, financial considerations, and perceived professional opportunities.

A review of the current literature shows that studies assessing the career plans of Saudi dental students at a national level remain scarce. A recent study appraising future career challenges among dental students and interns found that senior dental students perceive obtaining employment in the government sector, enrolling in a specialty program, and launching a private dental practice as significant future career challenges. 12 This study aims to investigate the future career plans of Saudi senior dental students and the factors shaping overall career plans. In addition, the determinants of interest in postgraduate studies will be evaluated.

## Materials and Methods

### Study Design and Participants

This study employed an online, questionnaire-based survey conducted following the STROBE guidelines. 13 A convenience non-randomized sampling method was used to recruit participants. The target study population comprised senior dental students in Saudi Arabia, defined as those in the final year of their dental program or undertaking the internship training year. Non-Saudi dental students were not eligible to participate.

### Sample Size Calculation

Sample size calculations were conducted using the Open-Source Epidemiologic Statistics for Public Health software, OpenEpi ( https://www.openepi.com/SampleSize/SSCohort.htm ). With a hypothesized outcome frequency of 50% (a standard estimate for unknown prevalence) and an absolute precision of 5%, a sample size of 384 participants was determined to be necessary for a 95% confidence interval. To account for potential missing data and non-responses, additional participants were included, resulting in a final sample size of 584.

### Data Collection Instrument

A self-administered questionnaire was developed following a comprehensive review of relevant literature. 1 3 7 14 The questionnaire, written in English, consisted of 25 items organized into three sections. The first section gathered demographic information from participants, including age, gender, academic level, type of university, grade point average (GPA), parents' educational level, and whether they had received guidance on career planning from their dental school. The second section focused on participants' career plans, including preferred future practice, desired employment sector, interest in postgraduate studies, specialty preferences, and intended retirement age. In the final section, participants rated the significance of 13 factors potentially influencing their career plans, using a five-point Likert scale ranging from “strongly agree” to “strongly disagree.”

A cover page introduced the questionnaire, outlining its purpose, objectives, and relevance. Participants were assured of confidentiality and anonymity in data handling and invited to consent to participate in the study.

To validate the questionnaire, a pilot study was conducted with 20 senior dental students to assess feasibility and clarity. Additionally, two senior academics, both PhD holders with over 10 years of academic experience, reviewed the instrument. One was a specialist in removable prosthodontics and the other in fixed prosthodontics. Feedback from the pilot study and the academic reviews was used to refine the final version of the questionnaire.

### Data Collection Procedures

The electronic version of the study questionnaire was created using the SurveyMonkey platform ( https://www.surveymonkey.com/welcome/sem/?program ). The survey link was disseminated via several WhatsApp and Instagram groups dedicated to dental students in Saudi Arabia, reaching ∼7,067 subscribers. Participation was restricted to Saudi final-year dental students and dental interns.

To ensure a representative sample from across the country, the authors targeted social media channels that included dental students from various dental institutions throughout Saudi Arabia. The survey remained open for over 6 months, with periodic reminders sent to eligible participants within these social media groups to maximize the response rate.

To maintain data integrity and prevent duplicate responses, the survey was configured to be IP address-sensitive, allowing only one submission per participant.

### Data Analysis

Data analysis was conducted using IBM SPSS Statistics for Windows, Version 25.0 (IBM Corp., Armonk, New York, United States). Descriptive statistics were applied to summarize participant demographics, and frequency tables were generated to display responses to survey questions.

To evaluate associations between questionnaire items and variables such as gender, GPA, and receipt of career guidance among senior dental students, the chi-square test was utilized. Statistical significance was set at a *p* -value of < 0.05.

Additionally, a multivariate binary logistic regression model was developed, with “interest in postgraduate studies” as the outcome variable. Predictor variables included gender, parents' education levels, GPA, type of university, and receipt of career guidance. Odds ratios (ORs) and 95% confidence intervals (CIs) were calculated to identify significant predictors of interest in postgraduate studies among Saudi senior dental students.

## Results

### Study Population

A total of 633 senior dental students participated in the study, of which 49 non-Saudi respondents were excluded. Consequently, the final study population consisted of 584 participants, comprising 205 final year Saudi dental students and 379 dental interns. However, the actual response rate could not be calculated, as the exact number of eligible students who received the invitation was unknown.

The data were collected from 12 dental schools across Saudi Arabia, encompassing 9 governmental and 3 private institutions. Notably, the majority of respondents (63.5%) reported receiving career plan guidance from their respective dental schools. Table 1 presents the characteristics of the study population.

**Table 1:** Characteristics of participants ( *N* = 584)

Age (years) Mean (SD): 24.5 (1.2) Range: 14 minimum 22 maximum 36	Gender Male: 361 (61.8%) Female: 223 (38.2%)	Academic level Final year: 205 (35.1%) Dental intern: 379 (64.9%)
**Type of university** **Public: 416 (71.2%)** **Private: 168 (28.8%)**	**GPA** 5–4.5: 152 (26%) 4.4–4: 302 (51.7%) <4: 130 (22.3%)	**Parents' educational level** Neither have a university degree: 97 (16.6%) One parent has a university degree: 142 (24.3%) Both parents have a university degree: 345 (59.1%)
**Received guidance on career planning from the dental school**		
** Yes: 371 (63.5%)**		
** No: 213 (36.5%)**		

Abbreviation: GPA, grade point average (out of five).

### Practice and Career Plans of Saudi Senior Dental Students

Table 2 illustrates the practice and career plans of Saudi senior dental students. The findings indicate that ∼50% of the participants aspired to become dental specialists, with a minority (11.3%) expressing a preference for working as general dental practitioners, and nearly 31% displaying interest in pursuing an academic career. Moreover, a significant majority of the participating dental students preferred employment in the government sector (63.2%), while only 12.8% favored the private sector. Additionally, an overwhelming majority of the students (87.5%) demonstrated interest in pursuing postgraduate studies. Among the surveyed students, endodontics emerged as the most preferred dental specialty (15.4%), followed by orthodontics (13.2%), periodontics (12.5%), and prosthodontics (12%). Conversely, family dentistry was the least preferred specialty among the study population (3.1%). Notably, a considerable number of participants (28.4%) planned to retire before the age of 50 years ( Table 2 ).

**Table 2:** Practice/career plans of Saudi senior dental students based on gender, GPA, and received guidance on career planning

Practice/career plans	Sample	Gender			GPA				Guidance on career planning		
		Male	Female	*p* -Value	5–4.5	4.4–4	<4	*p* -Value	Yes	No	*p* -Value
Preferred future practice/career plan											
General dental practitioner	11.3%	12.2%	9.9%	0.008 a	5.9%	7.6%	26.2%	<0.001 a	9.2%	15%	<0.001 a
Specialist dentist	49.3%	54%	41.7%		46.7%	55.6%	37.7%		54.7%	39.9%	
Academician/Teaching staff	11.3%	11.4%	11.2%		10.5%	12.9%	8.5%		15.4%	4.2%	
Clinician and academician	19.5%	15%	26.9%		26.3%	18.5%	13.8%		17.8%	22.5%	
Dental business	4.3%	4.2%	4.5%		4.6%	3.6%	5.4%		2.2%	8%	
Non-dental business	1.2%	1.1%	1.3%		0.7%	0	4.6%		0	3.3%	
Undecided	3.1%	2.2%	4.5%		5.3%	1.7%	3.8%		0.8%	7%	
Preferred sector for future work											
Governmental	63.2%	64.8%	60.5%	<0.001 a	65.8%	65.2%	55.4%	0.136	69.5%	52.1%	<0.001 a
Military	16.6%	23.8%	4.9%		11.8%	18.2%	18.5%		17%	16%	
Private	12.8%	7.5%	21.5%		13.2%	10.6%	17.7%		12.4%	13.6%	
Undecided	7.4%	3.9%	13%		9.2%	6%	8.5%		1.1%	18.3%	
Interested in postgraduate studies											
Yes	87.5%	87%	88.3%	0.364	94.1%	94.7%	63.1%	<0.001 a	95.4%	73.7%	<0.001 a
Practice/career plans	Sample	Gender			GPA				Guidance on career planning		
		Male	Female	*p* -Value	5–4.5	4.4–4	<4	*p* -Value	Yes	No	*p* -Value
Specialty of interest											
General dental practice	6.3%	8%	3.6%	0.193	3.9%	5.3%	11.5%	0.009 a	3.8%	10.8%	<0.001 a
Operative Dentistry	9.6%	9.4%	9.9%		13.8%	7%	10.8%		9.4%	9.9%	
Prosthodontics	12%	13.3%	9.9%		13.8%	10.9%	12.3%		13.2%	9.9%	
Endodontics	15.4%	16.6%	13.5%		20.4%	16.6%	6.9%		18.3%	10.3%	
Orthodontics	13.2%	11.1%	16.6%		9.9%	14.9%	13.1%		17%	6.6%	
Periodontics	12.5%	13%	11.7%		11.2%	13.6%	11.5%		13.5%	10.8%	
Oral surgery	7.5%	7.5%	7.6%		6.6%	9.3%	4.6%		7.8%	7%	
Oral medicine	10.4%	8.6%	13.5%		9.9%	11.3%	9.2%		9.7%	11.7%	
Family dentistry	3.1%	2.8%	3.6%		2.6%	3.3%	3.1%		2.7%	3.8%	
Pedodontics	5.3%	5%	5.8%		3.9%	5%	7.7%		3%	9.4%	
Undecided	4.6%	4.7%	4.5%		3.9%	3%	9.2%		1.6%	9.9%	
Planned age for retirement (years)											
< 50	28.4%	21.6%	39.5%	<0.001 a	30.3%	25.2%	33.8%	0.013 a	31%	23.9%	0.001 a
50–60	37.3%	37.4%	37.2%		32.9%	41.7%	32.3%		40.2%	32.4%	
> 60	7.2%	6.9%	7.6%		12.5%	4.3%	7.7%		4.6%	11.7%	
Undecided	27.1%	34.1%	15.7%		24.3%	28.8%	26.2%		24.3%	31.9%	

Abbreviation: GPA, grade point average (out of five).

a Significant difference at *p* < 0.05 as indicated by chi-square statistics.

Table 2 and Supplementary Table 1 (available in the online version only) further demonstrate significant differences in the future career plans of Saudi senior dental students, associated with gender, GPA, and career counseling by the dental school ( *p* < 0.05). A significantly higher number of male students (54%) planned to become specialist dentists compared with their female counterparts (41.7%; *p* < 0.05). Furthermore, a greater number of female dental students than males expressed plans to work both as clinicians and dental academics (26.9 vs. 15%; *p* < 0.05). Additionally, a significantly higher percentage of male students (23.8%) indicated a preference for working in the military sector compared with female students (4.9%). However, the preference of female students to work in the private sector exceeded that of their male peers (21.5 vs. 7.5%; *p* < 0.05). Moreover, a considerable number of female students (39.5%) planned to retire at an early stage in their careers (before the age of 50 years) in comparison with male students (21.6%; *p* < 0.05).

Regarding GPA, senior dental students with a GPA of less than 4 exhibited a higher preference for working as general dental practitioners, whereas those with a GPA of ≥4 expressed a greater interest in postgraduate studies ( *p* < 0.05). The impact of career counseling by the dental school was also significant, with students who received guidance showing a greater interest in postgraduate studies, a higher preference for becoming dental specialists or dental academics and a stronger preference for working in the government sector ( *p* < 0.05; Table 2 and Supplementary Table S1 [available in the online version only]).

### Factors Influencing Future Career Plans

Table 3 presents the attitudes of the senior dental students toward the impact of various factors on their future career plans. The most influential factor was “own personal desire,” with 95.7% of the participants strongly agreeing or agreeing on its importance. This was followed by the “reputation of a specialty/postgraduate program” (93.5%), and then “the needs/demands of the national job market” (92.8%). Notably, “the high chance of employment in the government sector” also received a high rating (91.1%). However, “parents' desire and surrounding community” attracted only 76.5%.”

**Table 3:** Factors that may influence Saudi senior dental students' future career plans

No	Factor	Participants' response ( *N* = 584)				
		Strongly agree	Agree	Undecided	Disagree	Strongly disagree
1	Own personal desire	74.1%	21.6%	3.6%	0.5%	0.2%
2	The parents' desire and the surrounding community	26.5%	50%	9.6%	5.8%	8%
3	The student's GPA	44.7%	43.5%	6.2%	3.3%	2.4%
4	The needs/demands of the national job market	57%	35.8%	6.2%	0.9%	0.2%
5	The needs/demands of the international job market	49.8%	36.1%	10.3%	3.1%	0.7%
6	The affordability of tuition/training fees	54.3%	36%	7%	1.7%	1%
7	The anticipated income	54.3%	38%	6.5%	1%	0.2%
8	The high chance of employment in the governmental sector	62.3%	28.8%	6.7%	2.1%	0
9	The high chance of employment in the private sector	57.2%	31.5%	8.2%	2.1%	1%
10	The nature of training in a specialty program	51.9%	40.4%	6.8%	0.7%	0.2%
11	The reputation of a specialty/postgraduate program	65.9%	27.6%	5.3%	1.2%	0
12	The flexible working time of a career/specialty	52.2%	37.8%	7.9%	1.7%	0.3%
13	The stress level of a career/specialty	56%	35.4%	5.3%	2.6%	0.7%

Abbreviation: GPA, grade point average.

### Predictors of Interest in Postgraduate Studies

To identify predictors of Saudi senior dental students' interest in postgraduate studies, a multivariate binary logistic regression model was employed. The model included gender, parents' education levels, GPA, type of university, and receipt of career guidance from the dental school. The ORs and their 95% CIs are presented in Table 4 . The results indicate that achieving a high GPA (OR for a GPA of 4–4.4 is 7.23 and for a GPA of 4.5–5 is 9.63), graduating from a public university (OR = 2.5), and receiving guidance on career plans from the dental school (OR = 6.33) are significant predictors of interest in postgraduate studies.

**Table 4:** Predictors of interest in postgraduate studies among Saudi senior dental students

	Odds ratio (OR)	95% confidence intervals	*p*
Gender			
Male	Ref	–	–
Female	1.45	0.79–2.66	0.23
Parents' education level			
Neither has a university degree	Ref	–	–
One parent has a university degree	1.68	0.73–3.86	0.22
Both parents have a university degree	1.99	0.98–4.03	0.06
GPA (out of 5)			
< 4	Ref	–	–
4–4.4	7.23	3.7–14.11	<0.001 a
4.5–5	9.63	4.24–21.86	<0.001 a
Type of the university			
Private	Ref	–	–
Public	2.5	1.39–4.55	<0.001 a
Received guidance on career planning from the dental school			
No	Ref	–	–
Yes	6.33	3.3–12.1	<0.001 a

Abbreviation: GPA, grade point average.

Note: The odds ratio and 95% confidence interval were calculated using a binary logistic model.

a Significant difference at *p* <0.05.

## Discussion

Several studies in Saudi Arabia have examined different aspects of dental students' careers, including reasons for choosing dentistry, 1 career satisfaction, 2 career motivations and perceptions of the future of dentistry, 3 career preferences and influencing factors, 7 potential challenges and opportunities, 9 12 and postgraduate interests and qualifications. 15 However, to our knowledge, no study has comprehensively evaluated the future career plans of Saudi senior dental students while simultaneously investigating the factors influencing their career decisions at a national level. Notably, this is the first study to identify predictors of interest in postgraduate education. By addressing these dimensions together, the present study provides new insights that can inform workforce planning and guide postgraduate training strategies in Saudi Arabia.

Career counseling is a crucial responsibility of academic institutions, aimed at ensuring that graduates are well-prepared for a smooth transition from academia to professional practice. It also equips them with the necessary knowledge and awareness to pursue further education, enhance their professional standards, and secure successful employment. In line with this perspective, a strategy and set of guidelines were developed in Canada to integrate career advice into the curricula of Canadian medical schools. Meeting these guidelines has been deemed essential for achieving the standards of academic accreditation in the Canadian medical education system. 16 In Saudi Arabia, the provision of effective professional guidance has become an essential standard for the accreditation of academic programs, due to the recognized cultural, educational, and economic benefits of career guidance. Research findings show that the Career Guidance and Counseling Program enhanced job readiness, confidence in career decisions, and adaptability to future career transitions among Saudi nursing students. 17 Moreover, a survey of Saudi pharmacy students found that although many colleges offered career counseling services, few students utilized them, and the lack of structured support was linked to gaps in career readiness. 18 In an earlier report, it was noted that most new labor market entrants have not received structured guidance at the university level on how to effectively plan and navigate their future career pathways. 19 In response, the Saudi Ministry of Labor has launched several initiatives to develop new policies and a career guidance system, drawing from global best practices. Additionally, the Ministry of Education and the General Directorate of Youth Organization have funded various career guidance projects. 19 In this study, 63.5% of participants indicated that they received guidance on career planning from their dental schools. While this figure is encouraging, it also highlights that a significant proportion of dental students in Saudi universities have not received formal career counseling. Greater efforts are needed to ensure that all dental students receive adequate and standardized career guidance, facilitating their smooth integration into the job market and postgraduate training programs.

A key finding of this study is that receiving career guidance from dental schools was a highly significant predictor of dental students' interest in pursuing postgraduate studies. The results show that a significantly higher proportion of senior dental students who received career guidance expressed interest in pursuing postgraduate studies and becoming specialist dentists, compared with those who did not receive such guidance. This highlights the positive impact of career guidance in motivating dental graduates to further their professional development. In practical terms, career guidance can be effectively integrated into postgraduate program planning by identifying students' interests early and tailoring curriculum components to meet these interests while addressing the specific needs and characteristics of the Saudi dental job market. This approach will better align students' career aspirations with market demands, thereby addressing the reported misdistribution and shortages within the dental workforce in Saudi Arabia. 4 6

Although it is unsurprising that achieving a higher GPA would predict a greater interest in postgraduate studies, the finding that graduates from public universities exhibit a stronger inclination toward postgraduate studies warrants further attention. One possible explanation is the high cost associated with dental education at private institutions, which may drive graduates toward general practice rather than pursuing additional academic qualifications. Further research is needed to investigate the underlying factors contributing to this trend and to assess the quality of career planning support provided by private dental schools in Saudi Arabia.

A study published in 2011 reported that most male graduates from King Saud University—College of Dentistry were motivated to pursue postgraduate education to enhance their careers and self-esteem. 15 In 2014, a study reported that 72.4% of a sample of Saudi dental students believed postgraduate education was essential for career advancement. 3 The author concluded that Saudi dental students' perceptions of the future of dentistry appeared to be linked to the need for pursuing postgraduate education. 3 Al-Hallak et al 1 reported that 94% of surveyed dental students expressed a willingness to enroll in postgraduate programs, projecting a rising demand for postgraduate dental education among Saudi graduates. The findings of our study align with this projection, as a significant majority of participants (87.5%) expressed interest in pursuing postgraduate studies. However, the finding that only 11.3% of dental students plan to work as general dental practitioners is concerning, as this may disrupt the balance between general practitioners and specialists, potentially exacerbating workforce shortages in primary dental care and limiting access to essential services, particularly in underserved areas. These results call for a comprehensive plan to accommodate the anticipated growing demand for postgraduate dental education and training programs in Saudi Arabia. Such a plan should also address any shortages in dental specialties across both urban and rural areas in various geographical regions, 4 while maintaining a balance between the number of general dental practitioners and specialist dentists. This context further emphasizes the importance of providing tailored career counseling.

Earlier studies have reported varying preferences among Saudi dental students regarding specializations. For instance, at King Saud University, male students predominantly preferred oral and maxillofacial surgery, followed by orthodontics, whereas female students showed a preference for operative dentistry, followed by pediatric dentistry. 3 A broader survey across 17 universities found restorative and esthetic dentistry to be the most popular choice, followed by endodontics and prosthodontics. 7 Al-Hallak et al 1 similarly reported orthodontics and oral surgery as top preferences. Pullishery et al 8 found that male dental interns in Saudi Arabia favored oral surgery and orthodontics, whereas female interns showed a preference for pediatric dentistry, oral medicine, and periodontics. In line with these findings, our study identified endodontics as the most favored specialty, followed by orthodontics, periodontics, and prosthodontics. Notably, a significantly higher proportion of senior dental students who reported receiving career guidance selected endodontics and orthodontics as their specialties of interest, compared with those who did not receive such guidance. This suggests that career guidance may play an influential role in shaping students' specialty choices. Strikingly, family dentistry emerged as the least preferred specialty (3.1%), despite its promising potential in the Saudi dental job market. These findings highlight both the diversity and evolving trends in specialty preferences, underscoring again the need for career guidance programs that align students' interests with market demands.

This study identified notable gender differences in the career and practice plans of male and female Saudi dental students, aligning with findings from prior research. 3 7 11 20 Interestingly, female students demonstrated a significantly higher preference than male students for careers in the private sector. A possible explanation for this trend may be the greater flexibility in working hours that private sector roles offer, which could be more compatible with the family obligations of Saudi female dentists. Understanding this trend is particularly important given the progressive increase in the number of female dentists and specialists in Saudi Arabia. 4 Moreover, with most dentists in Saudi Arabia employed in the private sector and only one-third working in the public sector, 4 these dynamics merit further consideration. In addition, the influence of career guidance on work sector choice should be considered, as our results indicate a significantly greater preference for employment in the government sector among students who received career guidance. Another notable gender difference relates to planned retirement age, with a considerable proportion of female participants (39.5%) intending to retire early (before the age of 50), compared with male participants (21.6%; *p* < 0.05). Family commitments for Saudi female dentists may again play a role in this decision. However, the potential future of early withdrawal or loss of a significant portion of the relatively young female dental workforce warrants attention and further investigation.

Understanding the factors that shape senior dental students' future career plans offers valuable insights for healthcare policymakers and workforce planners. Halawany et al 7 identified that “influence of family members in the dental profession,” “preference for private practice,” and “specific interest in patient population seen” were the three most critical factors affecting Saudi students' choice of specialty or general dentistry. In the present study, Saudi dental students indicated that their personal aspirations were the most influential factor in career decision-making, followed by the reputation of a specialty or postgraduate program and the needs and demands of the national job market as the second and third most influential factors, respectively. These findings suggest that Saudi senior dental students demonstrate maturity in making career and practice decisions, emphasizing the importance of incorporating these factors into Saudi career guidance programs.

Overall, this study demonstrates that the majority of senior dental students in Saudi Arabia have defined their career paths, with a strong interest in pursuing postgraduate education and a preference for employment in the government sector. Notable gender differences were observed, and the role of career counseling was found to be significantly influential in shaping students' career plans. With the current surplus of Saudi dental graduates, the growing pressure on the national dental job market and postgraduate education, and related imbalances and shortages in the dental workforce, the findings can inform dental education planners in Saudi Arabia and provide valuable insights for oral health service providers and Ministry of Labor authorities. Importantly, the results highlight the need for tailoring career guidance to better align students' aspirations with actual job market demands, thereby ensuring a more balanced workforce distribution. A further strength of this study is its inclusion of data from 12 dental colleges across Saudi Arabia, encompassing both public and private universities, and a robust sample size, which enhances the validity and generalizability of the findings. However, the use of a convenience sampling method and closed-ended questions, along with the lack of data from all dental programs in Saudi Arabia, may limit the study's broader applicability.

Future research should aim to incorporate a sample representative of all dental programs in Saudi Arabia, and the application of qualitative methods could yield a more comprehensive understanding of Saudi dental students' career aspirations. Research to explore the effectiveness of career guidance programs and their impact on graduates' career plans is highly recommended.

## Conclusion

Saudi dental students demonstrated diverse career aspirations, with a strong interest in postgraduate education and a preference for employment in the government sector. As career guidance has been identified as a significant predictor of students' interest in pursuing postgraduate studies, Saudi education, health, and labor authorities should seek to align students' career plans with the evolving needs and demands of the national job market to address potential imbalances in the dental workforce across the country.
