# Supplementary material for: Carving the Future: Career Aspirations of Senior Dental Students in Saudi Arabia
Source: Eur J Dent. 2025 Oct 28;20(3):1014–22. doi: 10.1055/s-0045-1812111 (PMC13337249; doi:10.1055/s-0045-1812111)
Supplement: Supplementary file 1 — Supplementary Material [file 10-1055-s-0045-1812111-s2564355-1.pdf]

Supplementary Table S1 Practice/career plans of Saudi senior dental students based on gender, GPA, and received guidance on career planning

Practice/career plans	Sample	Gender			GPA				Guidance on career planning		
		Male	Female	*p*-Value[a]	5-4.5	4.4-4	<4	*p*-Value[b]	Yes	No	*p*-Value[a]
Preferred future practice/career plan											
General dental practitioner	11.3%	12.2%	9.9%	0.389	5.9%	7.6%	26.2%	<**0.001**	9.2%	15.0%	**0.031**
Specialist dentist	49.3%	54.0%	41.7%	**0.004**	46.7%	55.6%	37.7%	0.18	54.7%	39.9%	<**0.001**
Academician/Teaching staff	11.3%	11.4%	11.2%	0.957	10.5%	12.9%	8.5%	0.64	15.4%	4.2%	<**0.001**
Clinician and academician	19.5%	15.0%	26.9%	<**0.001**	26.3%	18.5%	13.8%	**0.008**	17.8%	22.5%	0.164
Dental business	4.3%	4.2%	4.5%	0.849	4.6%	3.6%	5.4%	0.78	2.2%	8.0%	<**0.001**
Non-dental business	1.2%	1.1%	1.3%	0.798	0.7%	0.0%	4.6%	**0.004**	0.0%	3.3%	<**0.001**
Undecided	3.1%	2.2%	4.5%	0.123	5.3%	1.7%	3.8%	0.24	0.8%	7.0%	<**0.001**
Preferred sector for future work											
Governmental	63.2%	64.8%	60.5%	0.297	65.8%	65.2%	55.4%	**0.08**	69.5%	52.1%	<**0.001**
Military	16.6%	23.8%	4.9%	<**0.001**	11.8%	18.2%	18.5%	0.12	17.0%	16.0%	0.75
Private	12.8%	7.5%	21.5%	<**0.001**	13.2%	10.6%	17.7%	0.30	12.4%	13.6%	0.672
Undecided	7.4%	3.9%	13.0%	<**0.001**	9.2%	6.0%	8.5%	0.75	1.1%	18.3%	<**0.001**
Interested in postgraduate studies											
Yes	87.5%	87%	88.3%	0.629	94.1%	94.7%	63.1%	<**0.001**	95.4%	73.7%	<**0.001**
No/undecided	12.5%	13%	11.7%	0.629	5.9%	5.3%	36.9%	<**0.001**	4.6%	26.3%	<**0.001**
Specialty of interest											
General dental practice	6.3%	8.0%	3.6%	**.032**	3.9%	5.3%	11.5%	**0.011**	3.8%	10.8%	<**0.001**
Operative dentistry	9.6%	9.4%	9.9%	.858	13.8%	7.0%	10.8%	0.322	9.4%	9.9%	0.867
Prosthodontics	12.0%	13.3%	9.9%	.215	13.8%	10.9%	12.3%	0.665	13.2%	9.9%	0.23
Endodontics	15.4%	16.6%	13.5%	.303	20.4%	16.6%	6.9%	**0.002**	18.3%	10.3%	**0.01**
Orthodontics	13.2%	11.1%	16.6%	.056	9.9%	14.9%	13.1%	0.388	17.0%	6.6%	<**0.001**
Periodontics	12.5%	13.0%	11.7%	.629	11.2%	13.6%	11.5%	0.893	13.5%	10.8%	0.346
Oral surgery	7.5%	7.5%	7.6%	.949	6.6%	9.3%	4.6%	0.597	7.8%	7.0%	0.733
Oral medicine	10.4%	8.6%	13.5%	.062	9.9%	11.3%	9.2%	0.891	9.7%	11.7%	0.439
Family dentistry	3.1%	2.8%	3.6%	.579	2.6%	3.3%	3.1%	0.815	2.7%	3.8%	0.475
Pedodontics	5.3%	5.0%	5.8%	.659	3.9%	5.0%	7.7%	0.170	3.0%	9.4%	<**0.001**
Undecided	4.6%	4.7%	4.5%	0.9	3.9%	3.0%	9.2%	**0.046**	1.6%	9.9%	<**0.001**
Planned age for retirement (years)											
<50	28.4%	21.6%	39.5%	<**0.001**	30.3%	25.2%	33.8%	0.574	31.0%	23.9%	0.069
50–60	37.3%	37.4%	37.2%	0.966	32.9%	41.7%	32.3%	0.979	40.2%	32.4%	0.062
>60 years	7.2%	6.9%	7.6%	0.751	12.5%	4.3%	7.7%	0.087	4.6%	11.7%	**0.001**
Undecided	27.1%	34.1%	15.7%	<**0.001**	24.3%	28.8%	26.2%	0.692	24.3%	31.9%	**0.045**

[a] *p* was calculated based on chi-square.
[b] *p* was calculated based on chi-square for trend; GPA: grade point average (out of five).
Note: Bold *p*-values indicate a significant difference at $p < 0.05$.

 © 2025. The Author(s).
