# Supplementary material for: Carving the Future: Career Aspirations of Senior Dental Students in Saudi Arabia
Source: Eur J Dent. 2025 Oct 28;20(3):1014–22. doi: 10.1055/s-0045-1812111 (PMC13337249; doi:10.1055/s-0045-1812111)
Supplement: Supplementary file 2 — Supplementary Material [file 10-1055-s-0045-1812111-s2564355-2.pdf]

STROBE Statement—Checklist of items that should be included in reports of ***cross-sectional studies***

**Carving the future: Career aspirations of senior dental students in Saudi Arabia**

| Item No | Recommendation | Section in Manuscript |
|---|---|---|
| **Title and abstract** | | |
| 1a | Indicate the study's design with a commonly used term in the title or the abstract. | Abstract: "An online questionnaire-based survey, designed following STROBE guidelines, was conducted." (Implies cross-sectional as it's a survey at a point in time). |
| 1b | Provide in the abstract an informative and balanced summary of what was done and what was found. | Abstract/Done |
| **Introduction** | | |
| **Background/rationale** | | |
| 2 | Explain the scientific background and rationale for the investigation being reported. | Introduction: Paragraphs 1-6 |
| **Objectives** | | |
| 3 | State specific objectives, including any prespecified hypotheses. | Abstract: "Objectives: This study investigates the future career plans of senior dental students in Saudi Arabia and the factors influencing these plans."; Introduction: "...This study aims to investigate the future career plans of Saudi senior dental students and the factors influencing these plans." |
| **Methods** | | |
| **Study design** | | |
| 4 | Present key elements of study design early in the paper. | Methods: "This study employed an online, questionnaire-based survey conducted following the STROBE guidelines11." |
| **Setting** | | |
| 5 | Describe the setting, locations, and relevant dates, including periods of recruitment, exposure, follow-up, and data collection. | Methods: "The survey link was distributed through several WhatsApp and Instagram groups specifically for dental students in Saudi Arabia..."; "The survey remained open for over six months..."; Results: "The data were collected from 12 dental schools across Saudi Arabia, encompassing 9 governmental and 3 private institutions." |

| | | |
|---|---|---|
| **Participants** | | |
| 6a | Give the eligibility criteria, and the sources and methods of selection of participants. | Methods: "Participants included final-year dental students and interns from dental institutions across Saudi Arabia."; "The target population consisted exclusively of senior dental students enrolled in dental institutions in Saudi Arabia, specifically those in their final year of the dental program or those currently undertaking the internship training program."; "A convenience non-randomized sampling method was used to recruit participants." |
| **Variables** | | |
| 7 | Clearly define all outcomes, exposures, predictors, potential confounders, and effect modifiers. Give diagnostic criteria, if applicable. | Methods: "The second section focused on participants' career plans, including preferred future practice, desired employment sector, interest in postgraduate studies, specialty preferences, and intended retirement age."; "In the final section, participants rated the significance of 13 factors potentially influencing their career plans..."; Data Analysis: "To evaluate associations between questionnaire items and variables such as gender, GPA, and receipt of career guidance among senior dental students, the Chi-square test was utilized."; "Predictor variables included gender, parents' education levels, GPA, type of university, and receipt of career guidance." |
| **Data sources/ measurement** | | |
| 8* | For each variable of interest, give sources of data and details of methods of assessment (measurement). Describe comparability of assessment methods if there is more than one group. | Methods: "An online questionnaire-based survey..."; "A self-administered questionnaire was developed following a comprehensive review of relevant literature..."; "The questionnaire, written in English, consisted of 25 items organized into three sections."; "The electronic version of the study questionnaire was created using the SurveyMonkey platform..."<br>Data analysis describes comparability of assessment methods. |
| **Bias** | | |
| 9 | Describe any efforts to address potential sources of bias. | Methods: "To maintain data integrity and prevent duplicate responses, the survey was configured to be IP address-sensitive, allowing only one submission per participant." |
| **Study size** | | |

| | | |
|---|---|---|
| 10 | Explain how the study size was arrived at. | Methods: "Sample size calculations were conducted using the Open-Source Epidemiologic Statistics for Public Health software, OpenEpi..."; "With a hypothesized outcome frequency of 50%... and an absolute precision of 5%, a sample size of 384 participants was determined to be necessary for a 95% confidence interval."; "To account for potential missing data and non-responses, additional participants were included, resulting in a final sample size of 584." |
| **Quantitative variables** | | |
| 11 | Explain how quantitative variables were handled in the analyses. If applicable, describe which groupings were chosen and why. | Methods: "Descriptive statistics were applied to summarize participant demographics..."; "To evaluate associations between questionnaire items and variables such as gender, GPA, and receipt of career guidance among senior dental students, the Chi-square test was utilized."; "Additionally, a multivariate binary logistic regression model was developed, with 'interest in postgraduate studies' as the outcome variable. Predictor variables included gender, parents' education levels, GPA, type of university, and receipt of career guidance." GPA groupings (5 – 4.5, 4.4 – 4, <4) are used in Table 1 and Table 2. |
| **Statistical methods** | | |
| 12a | Describe all statistical methods, including those used to control for confounding. | Methods: "Data analysis was conducted using IBM SPSS Statistics for Windows, Version 25.0..."; "Descriptive statistics were applied to summarize participant demographics, and frequency tables were generated to display responses to survey questions."; "To evaluate associations between questionnaire items and variables such as gender, GPA, and receipt of career guidance among senior dental students, the Chi-square test was utilized."; "Additionally, a multivariate binary logistic regression model was developed... Predictor variables included gender, parents' education levels, GPA, type of university, and receipt of career guidance." |
| 12b | Describe any methods used to examine subgroups and interactions. | Methods: "To evaluate associations between questionnaire items and variables such as gender, GPA, and receipt of career guidance among senior dental students, the Chi-square test was utilized."; Results: "Table 2 and supplementary table further demonstrate that significant differences were |

| | | |
|---|---|---|
| | | observed in the future career plans of Saudi senior dental students associated with gender, GPA, and career counselling by the dental school ($p < 0.05$)." |
| 12c | Explain how missing data were addressed. | No missing data – The survey cannot be submitted unless all items are completed. |
| 12d | If applicable, describe analytical methods taking account of sampling strategy. | A convenience non-randomized sampling method was used. |
| 12e | Describe any sensitivity analyses. | NA. |
| **Results** | | |
| **Participants** | | |
| 13a | Report numbers of individuals at each stage of study—e.g., numbers potentially eligible, examined for eligibility, confirmed eligible, included in the study, completing follow-up, and analysed. | Results: "A total of 633 senior dental students participated in the study, of which 49 non-Saudi respondents were excluded."; "Consequently, the final study population consisted of 584 participants, comprising 205 final year dental students and 379 dental interns." |
| 13b | Give reasons for non-participation at each stage. | Results: "49 non-Saudi respondents were excluded." |
| 13c | Consider use of a flow diagram. | No flow diagram was used. |
| **Descriptive data** | | |
| 14a* | Give characteristics of study participants (e.g., demographic, clinical, social) and information on exposures and potential confounders. | Results: "Table 1 presents the characteristics of the study population..."; Table 1 (includes age, gender, academic level, type of university, GPA, parents' educational level, received guidance on career planning) |
| 14b | Indicate number of participants with missing data for each variable of interest. | NA |
| **Outcome data** | | |
| 15* | Report numbers of outcome events or summary measures. | Results: "Most respondents (63.5%) reported receiving career guidance, with 87.5% expressing interest in postgraduate studies and 11.3% preferring to work as general dental practitioners."; "A significant proportion (63.2%) favored employment in the government sector, while 12.8% preferred the private sector."; "Endodontics was the most preferred specialty (15.4%), followed by Orthodontics (13.2%), Periodontics (12.5%), and Prosthodontics (12%)."; "Additionally, 28.4% |

| | | |
|---|---|---|
| | | planned to retire before age 50." (Further details are in Table 2 and Table 3 ). |
| **Main results** | | |
| 16a | Give unadjusted estimates and, if applicable, confounder-adjusted estimates and their precision (e.g., 95% confidence interval). Make clear which confounders were adjusted for and why they were included. | Results: "Career plans were significantly associated with gender, GPA, and receipt of career guidance ($p < 0.05$)."; "Predictors of interest in postgraduate studies included a high GPA, graduation from a public university, and receiving career guidance from dental schools."; "The odds ratios and their 95% confidence intervals (95% CI) are presented in Table 4. The results indicate that achieving a high GPA (odds ratio for a GPA of 4-4.4 is 7.23 and for a GPA of 4.5-5 is 9.63), graduating from a public university (odds ratio = 2.5), and receiving guidance on career plans from the dental school (odds ratio = 6.33) are significant predictors of interest in postgraduate studies."; Table 4. Confounders (gender, parents' education levels, GPA, type of university, and receipt of career guidance) were included as predictor variables in the multivariate logistic regression model. |
| 16b | Report category boundaries when continuous variables were categorized. | GPA categories: "5 – 4.5", "4.4 – 4", "<4". Age categories for retirement: "< 50", "50-60", "> 60". |
| 16c | If relevant, consider translating estimates of relative risk into absolute risk for a meaningful time period. | Not relevant for this cross-sectional study. |
| **Other analyses** | | |
| 17 | Report other analyses done—e.g., analyses of subgroups and interactions, and sensitivity analyses. | Results: "Table 2 and supplementary table further demonstrate that significant differences were observed in the future career plans of Saudi senior dental students associated with gender, GPA, and career counselling by the dental school ($p < 0.05$)."; Specific gender differences are then detailed. Differences based on GPA and career counseling are also detailed. |
| **Discussion** | | |
| **Key results** | | |
| 18 | Summarise key results with reference to study objectives. | Discussion: "A key finding of this study is that receiving career guidance from dental schools was a highly significant predictor of dental students' |

| | | |
|---|---|---|
| | | interest in pursuing postgraduate studies."; "The findings of our study align with this projection, as a significant majority of participants (87.5%) expressed interest in pursuing postgraduate studies."; "our study identified endodontics as the most favored specialty, followed by orthodontics, periodontics, and prosthodontics."; "This study identified notable gender differences in the career and practice plans of male and female Saudi dental students, aligning with findings from prior research 3,7,16,17."; "In the present study, Saudi dental students indicated that their personal aspirations were the most influential factor in career decision-making, followed by the reputation of a specialty or postgraduate program and the needs and demands of the national job market as the second and third most influential factors, respectively."; "Overall, this study demonstrates that the majority of senior dental students in Saudi Arabia have defined their career paths, with a strong interest in pursuing postgraduate education and a preference for employment in the governmental sector." |
| **Limitations** | | |
| 19 | Discuss limitations of the study, taking into account sources of potential bias or imprecision. Discuss both direction and magnitude of any potential bias. | Discussion: "However, the use of a convenience sampling method and close-ended questions, along with the lack of data from all dental programs in Saudi Arabia, may limit the study's broader applicability." |
| **Interpretation** | | |
| 20 | Give a cautious overall interpretation of results considering objectives, limitations, multiplicity of analyses, results from similar studies, and other relevant evidence. | Discussion: Throughout the discussion section, the authors interpret their findings in the context of existing literature and the specific Saudi Arabian dental workforce situation. For example, regarding career guidance , GPA and public vs. private universities , interest in postgraduate studies vs. general practice , specialty preferences , gender differences and early retirement , and influential factors. |
| **Generalisability** | | |
| 21 | Discuss the generalisability (external validity) of the study results. | Discussion: "A further strength of this study is its inclusion of data from 12 dental colleges across Saudi Arabia, encompassing both public and private universities, and a robust sample size, which enhances the validity and generalizability of |

| | | |
|---|---|---|
| | | the findings."; "However, the use of a convenience sampling method and close-ended questions, along with the lack of data from all dental programs in Saudi Arabia, may limit the study's broader applicability." |
| **Other information** | | |
| **Funding** | | |
| 22 | Give the source of funding and the role of the funders for the present study and, if applicable, for the original study on which the present article is based. | This research received no funding. |
